# What's Behind Image? Toward a Better Understanding of Image-Driven Behavior

**DOI:** 10.3389/fpsyg.2021.614575

**Published:** 2021-06-09

**Authors:** Tobias Regner

**Affiliations:** Department of Economics, University of Jena, Jena, Germany

**Keywords:** social preferences, pro-social behavior, experiment, guilt, shame, reciprocity, self-image concerns, social-image concerns

## Abstract

Our experimental design systematically varies image concerns in a dictator/trust game. In comparison to the baseline, we either decrease the role of self-image concerns (by providing an excuse for selfish behavior) or increase the role of social-image concerns (by conveying the transfer choice to a third person). In this set up, we analyze the underlying processes that motivate subjects to give less/more. Controlling for distributional preferences and expectations, our results indicate that moral emotions (guilt and shame) are a significant determinant of pro-social behavior. The disposition to guilt explains giving in the baseline, while it does not when an excuse for selfish behavior exists. Subjects' disposition to shame is correlated to giving when their choice is public and they can be identified.

**JEL Classifications:** C72, C91, D03, D80

## 1. Introduction

What drives pro-social behavior, what motivates us to give more than we have to, even in non-repeated interactions? These questions led to a substantial body of academic work, the literature on social preferences^1^. Early models assumed distributional preferences as an explanation of other regarding behavior (Fehr and Schmidt, 1999; Bolton and Ockenfels, 2000; Andreoni and Miller, 2002), later on beliefs were incorporated into the utility function to take the effect of motives like reciprocity or emotions into account (Dufwenberg and Kirchsteiger, 2004; Falk and Fischbacher, 2006; Battigalli and Dufwenberg, 2007). Most recently, models emerged that consider the role of image as a determinant of pro-sociality (Akerlof and Kranton, 2000; Bodner and Prelec, 2003; Bénabou and Tirole, 2006, 2011; Ellingsen and Johannesson, 2008; Mazar et al., 2008; Andreoni and Bernheim, 2009; Grossman and van der Weele, 2017).

The insight of such self-/social-image models is that pro-social behavior can depend on the context. Exposure of our choice to others increases the chance of pro-social behavior (e.g., Ariely et al., 2009). Likewise, weakening the connection between action and the self-inducing moral wiggle room tends to decrease it as we are able to attribute the selfish action to the context, instead of having to connect selfish behavior to the self-image (e.g., Dana et al., 2007).

Our experimental study, a modified dictator game, sets out to test what are underlying psychological processes of image-driven behavior. We vary the extent of image concerns that may affect the transfer choice (by decreasing the role of self- and increasing the role of social-image of concerns). We also turn the dictator game into a trust one in order to study potential interaction effects between image and reciprocal concerns. This setup allows us to focus on determinants image-driven behavior, while we control for factors that are already known to motivate pro-social choices (distributional preferences and expectations).

Our results suggest that behavioral differences resulting from a variation of image concerns origin from moral emotions. The disposition to guilt determines transfers in the baseline but not when the connection between action and outcome is less clear. The disposition to shame is correlated with the transfer size only when another subject gets to know the transfer and potentially sees who made the transfer.

As our study offers a psychological foundation for image-driven behavior, we synthesize existing modeling approaches of social preferences. Belief-dependent models propose that psychological correlates like the disposition to guilt affect pro-sociality (in combination with expectations). Our results indicate that the role of moral emotions in explaining pro-social behavior goes beyond that. Moral emotions may be the responsible underlying process for behavior that has been attributed to image concerns.

A by-product of our design is an estimate of the relative explanatory power of the respective factors influencing pro-social behavior considered in our study. We find that the estimates of a one standard deviation change are very similar for the social value orientation, the second-order beliefs, the disposition to guilt, and the disposition to shame (between 1.06 and 1.17 with a mean transfer of 5.70).

The paper is organized as follows: In section 2, we describe the experiment and present behavioral predictions. Results are reported and discussed in section 3. Section 4 provides the conclusion.

## 2. Study

Our study consists of a lab experiment, which is preceded by an online survey that was administered through an Internet platform 1 week before the experiment.

### 2.1. Experimental Design

An allocation decision is at the core of the game played in the experiment. Three players are matched together. Player *Y* chooses how to divide 20 euros between himself and player *X* with every integer 0 ≤ *t* ≤ 20 possible as the transfer. A third player, *Z*, is passive and is not affected by the choice. Subjects know that the game is played just once. Our 4 ×2 between-subjects design varies the game along two dimensions: image (MorEx; baseline; Obs; ObsID) and reciprocity [dictator game (DG) vs. trust game (TG)].

In the *image* dimension, we change the extent of image concerns triggered by player *Y*'s choice. In the baseline and all other conditions, subjects know that player *X* only learns the received transfer at the very end of the experiment. In Obs, they are informed that player *Z* observes the transfer. In ObsID, subjects know that *Z* is informed about the transfer but also about the cubicle number of *Y*. Moreover, the instructions remind them that at the end of the experiment subjects are called to the front of the lab to receive their earnings. Thus, their cubicle number is announced before they walk to the front. While this is standard procedure in the lab, we also informed them that the order of paying the subjects is varied. It can be from cubicle 1 to 30, or decreasing from 30 to 1, or from 15/16 going down-/upward. Hence, they are made aware that irrespective of their actual cubicle number, a player *Y* can be seen by his/her *Z*. Finally, in MorEx, subjects are informed that there is a 50% chance of nature overwriting *Y*'s choice. In that case, the computer picks any possible transfer with equal chance. While *Y* is told whether his decision is implemented or not, *X* does not find out whether the received transfer is *Y*'s choice or overwritten by the computer. This setup offers a moral/situational excuse for behaving selfish by introducing uncertainty. See Exley (2015) or Regner and Matthey (2017) for similar designs.

In *reciprocity*, the game is either played as it is (dictator) or with a preceding stage (trust) in which player *X* has a binary choice between entering the game (and letting *Y* decide) and an outside option that results in a payoff of 5 euros for both *X* and *Y*. We ask trustees for their decisions independent of the trustor's choice, that is, we use the strategy method (Selten, 1967).

To summarize, our experimental design systematically varies image concerns by decreasing self-image concerns in MorEx (with social-image concerns kept constant) and by increasing social-image concerns in Obs and ObsID (while self-image concerns remain constant). See Table 1 for an overview of the image treatments and their features. We also compare a dictator to a trust game setting in order to study potential interaction effects between image and reciprocal concerns.

**Table 1:** Overview of image treatments and their features.

**Image treatment**	**MorEx**	**Baseline**	**Obs**	**ObsID**
50% chance of overwriting	Yes	No	No	No
*Z* observes transfer	No	No	Yes	No
*Z* informed about *Y*'s cubicle number	No	No	No	Yes

After game choices were made, we asked subjects for their probabilistic (or distributional) first- and second-order *beliefs*. For subjects *Y*, this is the belief with respect to *X*'s choice to enter the game (first-order belief), and the belief about the expectation of subject *X* with respect to subject *Y*'s transfer (second-order belief). For subjects *X*, this is the belief about subject *Y*'s transfer (first-order belief), and the belief about the expectation of subject *Y* with respect to subject *X*'s choice to enter the game (second-order belief). Subject *Z* was asked for two first-order beliefs (with respect to *X*'s choice to enter the game and about *Y*'s transfer) and two second-order beliefs (about the expectation of *X* with respect to *Y*'s transfer and about the expectation of *Y* with respect to *X*'s choice to enter the game).

The probabilistic beliefs were collected as vectors for a series of intervals. Regarding the choice to enter the game, subjects could assign their first-order belief to the options 0 (no) and 1 (yes) and the second-order belief to the intervals [0, 10), [10, 20), [20, 30), ..., [90, 100] percent. They could distribute their belief regarding the transfer to the following intervals: [0, 2), [2, 4), [4, 6), ..., [18, 20] euro. The software made sure that the numbers a subject is assigned sum up to 100%. Figure 2 in Appendix A shows a screenshot of the decision interface for the first-order belief of a player *X* (or *Z*). Beliefs were elicited using a quadratic scoring rule. In contrast to a linear scoring rule, a quadratic one is incentive compatible which tends to result in more accurate predictions (see Palfrey and Wang, 2009; Armantier and Treich, 2013; Schotter and Trevino, 2014). In contrast to point (or non-probabilistic) forecasts, probabilistic forecasts allow participants to express uncertainty about their belief. See Manski and Neri (2013) for a comprehensive account of probabilistic and non-probabilistic elicitation of second-order beliefs.

The instructions informed subjects that after stage 1 of the experiment, consisting of the game as described, they will play two more stages for which they receive instructions in due course. In stage 2, the same game was played but roles were changed: subjects were rotated, that is, player *Y* of stage 1 now played as *X*, *Z* as *Y*, and *X* as *Y*. In stage 3, subjects were rotated once more so that each subject played in each role. Resulting payoffs of all stages were only announced at the end of the experiment (after stage 3). Subjects knew that one of the three stages was randomly chosen as payoff-relevant.

### 2.2. Measures From the Online Survey

A week before the actual lab experiment, subjects participated in an online survey administered through an Internet platform. The aim of the survey was to assess subjects' *social value orientation* and their *dispositions with respect to guilt and shame* in advance of the actual experiment.

The *social value orientation* (SVO) slider measure (Murphy et al., 2011) consists of six primary items and nine optional ones. In each item, subjects face a resource allocation choice over a well-defined continuum of payoffs for themselves and someone else: for instance, one item features a trade-off between the perfectly altruistic choice of (50, 100) and the perfectly individualistic choice of (100, 50). In between these extreme values, there are always seven allocations that allow for intermediate choices. From choices in the six primary items, the SVO angle is computed, a continuous measure that we employ as a proxy for the subjects' concern for the payoff of others. The SVO angle reflects individualistic (maximizing own payoffs), competitive (maximizing the difference between own and other's payoff), inequality averse (minimizing the difference between own and other's payoff), and efficiency (joint payoff maximizing) motives.

The Guilt And Shame Proneness scale (GASP) by Cohen et al. (2011) is an innovative scale to measure individuals' *dispositions with respect to guilt and shame*. It assumes that private transgressions trigger feelings of guilt, while public transgressions trigger feelings of shame. Hence, their guilt scenarios are all set in the private domain, and the shame scenarios are always public situations. It also incorporates the self-behavior conceptualizations of shame and guilt and additionally distinguishes evaluative responses from action orientations. In total, the GASP consists of 16 real-life scenarios. Subjects are asked to imagine they were in that situation and indicate the likelihood that they would react in the way described at the end of the scenario^2^. While the ability to evaluate own behavior (captured by the NBE sub-scale) should be most indicative for pro-social guilt-driven behavior, the evaluative sub-scale for shame (NSE) should be indicative for an ability to anticipate feeling ashamed.

### 2.3. Behavioral Predictions

The literature of social preferences started off with outcome-based models (Fehr and Schmidt, 1999; Bolton and Ockenfels, 2000; Andreoni and Miller, 2002) using distributional preferences in order to explain pro-social behavior. Subsequently, with the development of belief-dependent models (Dufwenberg and Kirchsteiger, 2004; Falk and Fischbacher, 2006; Battigalli and Dufwenberg, 2007), the role of expectations as a determinant of pro-social behavior gained attention.

In line with this literature, we generally expect that two factors motivate the choice of the transfer in our experiment: the individual's distributional preferences and expectations about what the recipient expects to get. Thus, we expect that the size of the transfer is positively correlated with our proxy for the level of distributional preferences, the SVO angle, and the second-order beliefs^3^ of the player who sends the transfer.

More recently, image concerns have been incorporated in the economic modeling of pro-social behavior. *Self-image* concerns (Murnighan et al., 2001; Bodner and Prelec, 2003; Mazar et al., 2008; Bénabou and Tirole, 2011) explain pro-social behavior as a consequence of desiring a self-image (alternatively, a self-concept or behavioral standard) of not being selfish^4^. As deviating from the pro-social self-image is psychologically costly, selfish choices result, only if the monetary gain of a selfish action outweighs that cost. Supporting the relevance of self-image concerns, a series of studies, started by Dana et al. (2007), finds that pro-social choices are significantly reduced when moral excuses for selfish behavior are available. Evidence of such “moral wiggle room” indicates/suggests that the effect of self-image concerns is toned down, if the connection between actions and the self is blurred. Once individuals are able to attribute their selfish action to the context, instead of having to connect selfish behavior to their self-image, they tend to behave more selfish.

Individuals can also have *social-image* concerns (Bénabou and Tirole, 2006; Ellingsen and Johannesson, 2008; Andreoni and Bernheim, 2009), if they desire not to appear selfish to others, especially their peers. Due to such concerns for their social reputation, individuals would be more likely to make a pro-social choice, if an audience they care about is able to observe their decision. Plenty of empirical evidence from the lab (e.g., Kurzban et al., 2007; Ariely et al., 2009; Henry and Sonntag, 2019) and the field (e.g., Lacetera and Macis, 2010; Bursztyn and Jensen, 2017) highlights the importance of the social image component when it comes to pro-social behavior.

Our experimental design systematically varies image concerns. In comparison to the baseline, the treatment MorEx decreases the role of self-image concerns as it provides a moral excuse for selfish behavior. As subjects know that the transfer may be overwritten, players *Y* may send a low amount, and *X* cannot distinguish whether it was *Y*'s choice or forced by the computer. Moreover, they know that their choice of a low transfer may not actually matter as it could be replaced by the computer^5^. Thus, we expect that transfers tend to be smaller.

**Hypothesis 1**. *When moral excuses are available (MorEx), transfers are, on average, lower than in the baseline.*

In comparison to the baseline, treatments Obs and ObsID increase the role of social-image concerns as the transfer choice is conveyed to a third person. Thus, selfish behavior potentially bears a reputational cost. We consider treatment Obs as a weak manipulation of social image (public exposure) though, since due to the anonymity of the experiment the choice cannot be traced back to a specific subject. This anonymity is lifted in treatment ObsID. Subjects know that their observer (player *Z* when they played as *Y*) is not only informed about the transfer but also might well be able to identify him-/herself at the end of the experiment. Note that other dimensions of social-image concerns are constant across conditions. Players *X* always know what they receive but never find out who sent it. The experimenter sees the subjects when handing over their payoffs but does not know the stage and role of the subject.

**Hypothesis 2**. *When the choice can be observed (Obs, ObsID), transfers are, on average, higher than in the baseline.*

Our next hypothesis addresses the interplay between image concerns and reciprocity. Some studies already investigated self-image concerns in the context of reciprocity. Regner and Matthey (2017) and Regner (2018a) find that the effect of moral wiggle room prevails in the context of reciprocity, while van der Weele et al. (2014) do not^6^. Ellingsen and Johannesson (2008) propose that social-image concerns depend on the audience; people care relatively more about the approval of peers, of people who are like them. Trustors' behavior (entering the game vs. outside option) can be seen as a signal about their pro-sociality which would tend to affect the concern trustees have for them. In Obs and ObsID, having been trusted in the first place may increase the level of approval toward the trustor and potentially amplifies the positive effect of social-image concerns on behavior. Hence—assuming reciprocal behavior in the baseline—we test whether reciprocity is more pronounced when an audience exists.

**Hypothesis 3**. *The difference between transfers in trust and dictator is, on average, higher in a public context (Obs and ObsID) than in the baseline.*

Given that image concerns affect behavior across treatments—on top of the effect of beliefs and SVO—we are also interested in the processes behind this relationship. An aversion to experience guilt, as proposed by Battigalli and Dufwenberg (2007), can be a determinant of the allocation decision: the more I believe you were disappointed due to my choice, the more guilt I would anticipate to feel. According to Tangney (1995) individuals differ in the degree to which they are prone to feel guilt. Thus, expectations as well as the sensitivity of a person to experience guilt influence the choice, and a series of empirical evidence supports these relationships (e.g., Charness and Dufwenberg, 2006; Pelligra, 2011; Bracht and Regner, 2013; Khalmetski, 2016; Cartwright, 2019). In our baseline, the relationship between the choice of *Y* and what *X* receives is transparent. Therefore, we expect the subjects' disposition to guilt to be positively correlated with the size of the transfer. However, the introduced uncertainty in MorEx means that subjects do not have to link the outcome of the recipient to their choice. They can tell themselves that even though *X* might be disappointed by the chosen transfer, there is still a 50% chance that their choice does not count^7^. Hence, we expect a breakdown of the relationship between the subjects' disposition to guilt and the size of the transfer in MorEx.

**Hypothesis 4**. *The GASP NBE (disposition to guilt) is a positive determinant of the transfer in the baseline (and Obs) while it is not in MorEx.*

Next, we look at social-image concerns in more detail. What are the potential underlying processes behind increased pro-social behavior in a public context? Based on insights from social psychology (e.g., Combs et al., 2010; Wolf et al., 2010), transgressions of morally accepted behavior in the public trigger feelings of shame (while transgressions that remain within the self lead to feelings of guilt).

In the context of our experiment, the morally accepted behavior is arguably an even split, that is, a transfer of 10. The more a subject falls short of that amount, the higher the resulting transgression might be. Hence, we expect that in ObsID subjects' disposition to shame is, on average, positively correlated with the transfer as they anticipate that a low transfer might result in a shameful experience.

**Hypothesis 5**. *The GASP NSE (disposition to shame) is a positive determinant of the transfer in ObsID.*

### 2.4. Participants and Procedures

We recruited 240 subjects from various disciplines at the local university using ORSEE (Greiner, 2004). In each session, gender composition was approximately balanced and subjects took part only in one session. Subjects who already participated in similar experiments were excluded from the recruitment pool. The experiment was programmed and conducted with the software z-Tree (Fischbacher, 2007) and took, on average, 60 min. The average earnings in the experiment have been €12.69 (plus a €2.50 show-up fee and €3 for completing the online survey). Only subjects who completed the online survey were allowed to participate in the experiment. However, one subject slipped through the controls, and survey data are not available.

Upon arrival at the laboratory, subjects were randomly assigned to one of the computers. Each computer is in a cubicle that does not allow communication or visual interaction. After subjects finished reading the instructions, they were asked to answer a set of control questions in order to ensure understanding. After all subjects had answered the questions correctly, the experiment started. At the end of the experiment subjects were paid in cash according to their performance. Privacy was guaranteed during the payment phase.

## 3. Results

We start with some descriptives of the data and proceed then to regression analyses in order to test our hypotheses.

**Figure 1:**
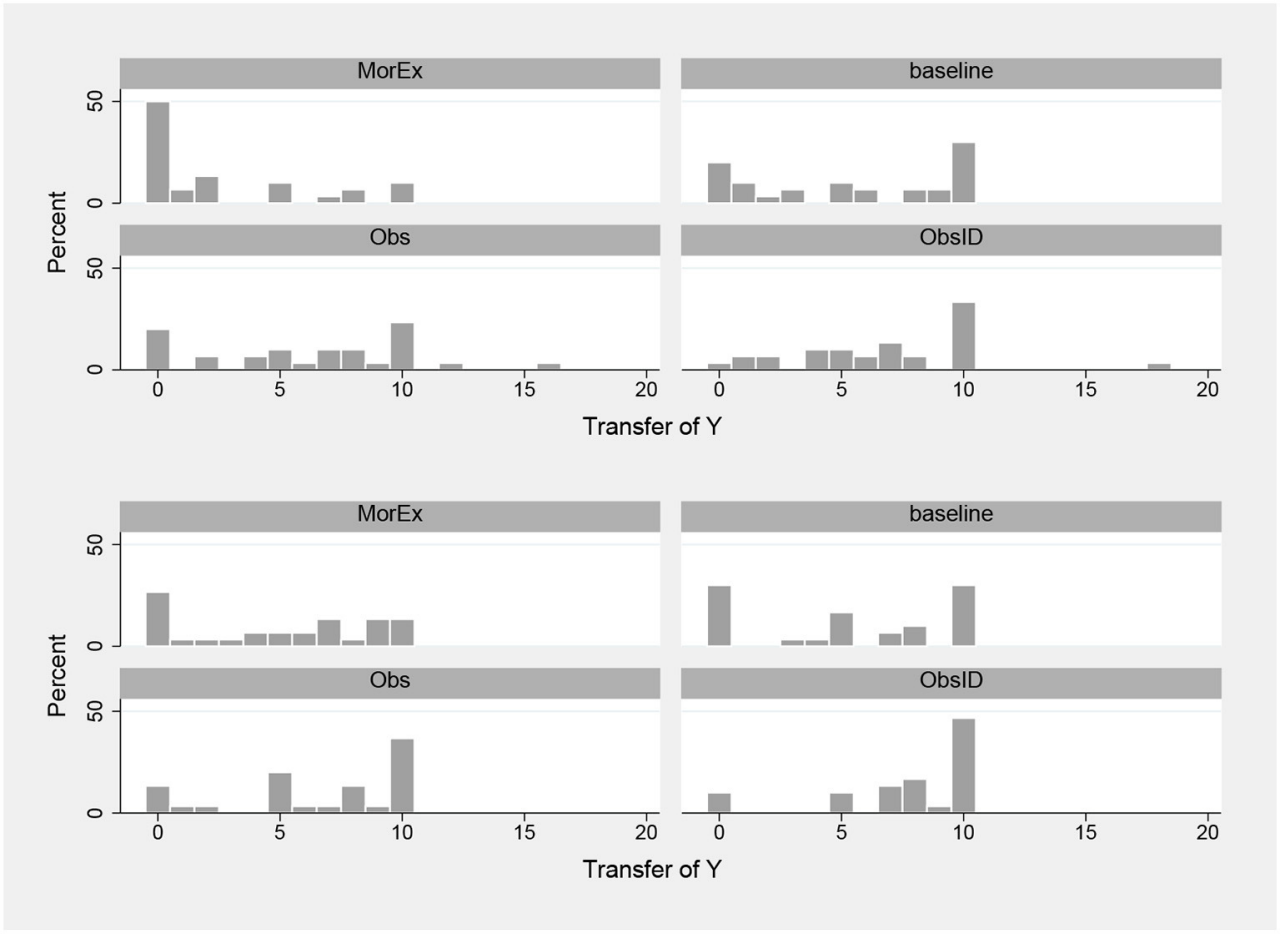
Histograms of transfers by treatment (top: dictator; bottom: trust).

Figure 1 shows histograms of the transfer for each treatment. Transfers increase along the image dimension of our design [means are 3.77 (MorEx), 5.37 (baseline), 6.37 (Obs), 7.32 (ObsID)] and the histograms give an indication why. In MorEx, the distributions peak at zero. The ones in the baseline are bi-modal, featuring a spike at zero and one at the equally splitting transfer of ten. This is also the case in Obs for the dictator condition, while in the trust condition and in ObsID, the spike at zero disappears.

Table 2 presents the results of OLS regressions with robust standard errors. The dependent variable is the transfer *Y* sends to *X*. The specification in the first column includes dummies for the treatments (TG represents the trust condition) and a control for the stage as some subjects played as *Y* in stage 1, some in 2, and some in 3. The dummy for MorEx is negative and significant at the 5%-level, the dummy for Obs is not significantly different from zero, and the dummy for ObsID is positive and significant at the 1%-level. The specification in column 2 adds the SVO angle and second-order beliefs as further control variables. Their coefficients are positive and highly significant, while the significance levels of MorEx and ObsID drop. The specification in column 3 adds an interaction term between TG and the second-order beliefs. The dummy for TG is positive and significant at the 5%-level, while the interaction term between TG and the second-order beliefs is negative and significant at the 5%-level. The dummy for ObsID is significant at the 5%-level, and the dummy for MorEx is still negative but not at a significant level. Finally, specification 4 adds interaction terms between the trust condition and the image dummies in order to test for the effect of reciprocity. Neither the main effects nor the interaction terms are significant. Dropping the controls, SVO angle and second-order beliefs deliver the same qualitative results.

**Table 2:** Treatment comparison.

	**(1)**	**(2)**	**(3)**	**(4)**
MorEx	−1.60^**^ (0.71)	−1.07^*^ (0.62)	−0.99 (0.63)	−1.98^**^ (0.86)
Obs	1.00 (0.73)	1.00 (0.64)	1.00 (0.63)	0.91 (0.83)
ObsID	1.95^***^ (0.68)	1.49^**^ (0.58)	1.49^***^ (0.57)	0.74 (0.76)
TG	0.88^*^ (0.49)	0.46 (0.45)	2.44^**^ (0.97)	1.53 (1.24)
Stage	−0.66^**^ (0.31)	−0.46^*^ (0.27)	−0.46^*^ (0.27)	−0.46^*^ (0.27)
SVO angle		0.10^***^ (0.021)	0.10^***^ (0.021)	0.10^***^ (0.021)
2nd order beliefs		0.30^***^ (0.075)	0.44^***^ (0.10)	0.43^***^ (0.11)
TG × 2nd order beliefs			−0.29^**^ (0.13)	−0.29^**^ (0.14)
MorEx × TG				2.01 (1.25)
Obs × TG				0.17 (1.24)
ObsID × TG				1.53 (1.14)
Constant	6.24^***^ (0.83)	1.62^**^ (0.78)	0.84 (0.83)	1.30 (0.92)
Observations	240	239	239	239
*R*^2^	0.136	0.348	0.362	0.373

*OLS regressions; robust standard errors in parentheses;*

* *p < 0:10,*

** *p < 0:05,*

*** *p < 0:01; one subject managed to participate in the experiment without having completed the online survey; variance inflation factors are all <2, indicating no concern for multicollinearity*.

Our results are consistent with our expectation that the SVO angle and second-order beliefs are significant determinants of the transfer. Furthermore, they support our hypothesis that transfers are higher when social-image concerns matter, albeit only in the treatment with potential public identification (ObsID). Also, our hypothesis regarding MorEx—transfers are lower when self-image concerns are inhibited by an available excuse—finds support although the effect is weakened when adding controls. While we find general reciprocal behavior (positive and significant coefficient of TG in specification 3), the effect in the baseline is not strong enough to be significant and neither are the interactions between the TG and other treatment dummies (specification 4). The apparent lack of baseline reciprocity complicates the testing of the respective hypothesis (increased reciprocal behavior in treatments Obs and ObsID), and we will get back to this later. Finally, the negative interaction effect between TG and second-order beliefs (in combination with their positive main effects) indicates that either the mere fact of being in the trust condition or second-order beliefs increase transfers in TG but not both factors jointly.

**Result 1**. *In MorEx, transfers are, on average, lower than in the baseline.*
**Result 2**. *In ObsID, transfers are, on average, higher than in the baseline.*

We proceed to a more detailed analysis of image-driven behavior. For this purpose, Table 3 presents one OLS regression for each image condition. The dependent variable is again *Y*'s transfer. Explanatory variables are dummies for the TG condition, the SVO angle, second-order beliefs, and the two sub-scales from the GASP that proxy the disposition to guilt (NBE) and shame (NSE).

**Table 3:** Processes within each image condition.

	**MorEx**	**Baseline**	**Obs**	**ObsID**
TG	2.29 (1.79)	2.86 (1.91)	4.88^**^ (2.22)	1.86 (2.30)
SVO angle	0.13^***^ (0.040)	0.081^**^ (0.034)	0.091^**^ (0.041)	0.13^***^ (0.028)
2nd order beliefs	0.24 (0.19)	0.67^***^ (0.17)	0.65^**^ (0.25)	0.31^*^ (0.16)
TG × 2nd order beliefs	−0.0041 (0.26)	−0.48^*^ (0.25)	−0.77^**^ (0.31)	−0.095 (0.27)
GASP_NBE	−0.46 (0.48)	0.95^**^ (0.40)	−0.22 (0.45)	−0.76^*^ (0.38)
GASP_NSE	0.80 (0.62)	−0.64 (0.46)	0.74 (0.53)	1.13^***^ (0.41)
Stage	−0.052 (0.55)	−0.33 (0.51)	−0.33 (0.59)	−0.78^*^ (0.44)
Constant	−3.38 (3.19)	−1.54 (2.66)	−2.05 (3.90)	0.55 (2.73)
Observations	59	60	60	60
*R*^2^	0.332	0.477	0.251	0.449

*OLS regressions; robust standard errors in parentheses;*

* *p < 0:10,*

** *p < 0:05,*

*** *p < 0:01; variance inflation factors are all <5, indicating no concern for multicollinearity*.

In MorEx, only the SVO angle is significant (at the 1%-level). In the baseline, the SVO angle and second-order beliefs are significant. In addition, the NBE sub-scale is significant at the 5%-level. Results in Obs resemble the overall results presented in Table 2: SVO angle, second-order beliefs, TG, and the interaction term between the last two are significant. In ObsID, the SVO angle and the NSE sub-scale are significant at the 1%-level.

Results in MorEx and the baseline support the respective hypotheses. With full transparency between actions and outcomes, the moral compass of subjects seems to be intact. As subjects know their transfer potentially disappoints *X*, anticipated guilt seems to keep them from sending low amounts, in line with the results of Bracht and Regner (2013). In contrast, when a low transfer choice does not necessarily mean a small received amount, the beliefs/guilt/pro-sociality system appears to break down. Only a base level of pro-sociality remains in the data.

**Result 3**. *The disposition to guilt is positively correlated to the transfer in the baseline but not in MorEx.*

Also, results in ObsID are consistent with our corresponding hypothesis. The disposition of the subjects to shame is a significant determinant of their transfer, when the setting is public and they could be recognized by the person who is informed about their transfer. The shame effect appears to crowd out the effect of second-order beliefs and of the TG treatment. In an additional specification, we included an interaction term between the second-order beliefs and the disposition to shame. The interaction is not significant. The effect of shame seems to stand on its own. This seems to suggest that the effect of shame (and anticipating it) is not about someone else and their expectations but the self.

**Result 4**. *The disposition to shame is positively correlated to the transfer in ObsID but not in baseline.*

Based on the treatment-specific coefficients shown in Table 3, the following changes of the estimated transfer result from a one standard deviation change of our main explanatory variables: SVO angle (1.06–1.7), second-order beliefs (1.06–2.29), GASP NBE (1.17), and the GASP NSE (1.16). Ranges express minimum/maximum values when the coefficient is significant.^8^ Given that the mean transfer is 5.70, a one standard deviation change results in roughly a 20% variation of the transfer (using lower bound estimates), independent of which factor changes. Hence, our statistically significant results also seem economically relevant.

Our results so far show that behavioral differences resulting from a variation of image concerns appear to have a sound psychological foundation in moral emotions. The disposition to guilt—in combination with expectations—determines transfers in the baseline but not in MorEx when the connection between action and outcome is less clear. The disposition to shame is correlated with the transfer size in ObsID when another subject gets to know the transfer and potentially sees who made the transfer. Increased pro-social behavior under public exposure is in line with results in Tadelis (2011). In his experiment, trustees cooperate significantly more often when their choice is announced to the entire lab than in the baseline. He does not elicit subjects' disposition to shame, though. Our results are less clear with respect to the interplay between image concerns and reciprocity (hypothesis 3). We do find overall reciprocal behavior (after controlling for the SVO angle, second-order beliefs, and their interaction with the trust dummy), but the effect is not significant in the baseline alone. Moreover, there is no evidence of increased reciprocity in the treatments with a public context (Obs and ObsID). A possible explanation is that our manipulation can be regarded as relatively weak. We use the strategy method for trustors' choices and, therefore, trustees do not know for sure whether they are trusted or not^9^. The actual effect of the trust condition on the transfer may, however, be affected by the beliefs of the subjects.

We turn to our beliefs data in an attempt to shed more light on this. Figure 3 in Appendix E shows the distribution of second-order beliefs (see Table 6 in Appendix D for summary statistics). Recall that we elicited probabilistic beliefs, not just point beliefs. That is, each subject told us the distribution of their beliefs, allocating probability weights to 10 intervals. Thus, Figure 3 in Appendix E illustrates the average weights, across subjects, for all intervals. Generally, in MorEx-dictator, subjects express the most pessimistic second-order beliefs. Most strikingly, about 33% of the probability mass is, on average, assigned to the interval including a first-order belief of a transfer of zero. In contrast, this is the case for only about 13% in MorEx-trust (ranksum test, *p* = 0.006). In baseline, this pattern is similar but less pronounced (about 26% in baseline-dictator vs. 13% in baseline-trust, ranksum test, *p* = 0.06). Beliefs in Obs and ObsID tend to be more optimistic in trust than in dictator, although beliefs of a zero transfer are practically equal. Overall, it seems that the trust condition has a positive effect on the subjects' second-order beliefs, which are positively correlated with the transfer. Thus, testing for the true effect of the trust condition in public settings would require to take second-order beliefs into account. Indeed, results of a mediation analysis indicate that the effect of the trust condition on the transfer is partly mediated by second-order beliefs^10^. Therefore, our overall results suggest that besides the direct effect of the trust condition on the transfer, there exists an indirect effect *via* higher second-order beliefs.

## 4. Conclusions

Our experiment systematically varies the role of image concerns in order to study the underlying processes that determine pro-social behavior. In comparison to our baseline, our design reduces the role of self-image concerns by providing a moral excuse for selfish behavior in the MorEx condition, and it allows for social-image concerns by introducing an audience in conditions Obs and ObsID.

We find that behavior across the conditions is in line with image concerns: Transfers are lower in MorEx and higher in ObsID. Our further analysis provides a psychological basis for image-driven behavior. We show that the disposition to guilt, a known determinant of pro-social behavior in previous research and also significant in our baseline, does not guide subjects when a moral excuse exists. Under public exposure of the transfer and potential facial identification of the subject who made the transfer, the disposition to shame is a significant determinant of the transfer choice.

Thus, our results suggest that moral emotions, like guilt and shame, are an important driver behind context-dependent pro-sociality^11^. Does that mean our pro-social choices are “emotional,” rooted in system 1? Two recent meta-studies analyze the role of intuition and deliberation in cooperation. While Fromell et al. (2020) find no significant difference when the intuitive system 1 was promoted at the expense of the deliberative system 2, Rand (2016) reports a 17% increase of “pure” cooperation when intuition was promoted over deliberation. It is the anticipation of guilt/shame that is behind pro-social choices in the belief-dependent models. Such an active avoidance of a potentially unpleasant situation arguably requires deliberation while emotions are involved at the same time. Thus, it is not necessarily an intuitive action, yet one based on emotions.

Furthermore, we find that our proxy for distributional preferences—the SVO angle—is a consistent determinant of the transfer size across all treatments. Also second-order beliefs—the key parameter of belief-dependent models—are a significant explanatory factor of the transfer. Interestingly, all four factors seem to have a similar impact on the size of the transfer (a one standard deviation change results in roughly a 20% variation).

It seems that distributional preferences, expressed by the SVO angle in our setting, provide a base level of pro-sociality that is unaffected by our treatment manipulations. Beliefs about others' expectations appear to play a major role in determining pro-sociality in treatments without (successful) manipulation. If the connection between choice and outcome is manipulated to be less transparent, the positive influence of second-order beliefs (and the disposition to guilt) on the size of the transfer erodes. Likewise, second-order beliefs seem to play only a marginal role when our treatment manipulation allows for public identification of the transfer and who sent it. The positive effect of the disposition to shame, a self-focused construct, appears to crowd out the impact of beliefs about others.

To conclude, we discuss the limitations and the possible future expansions of our study. One potential concern about our results is that the sample size per treatment cell (30 subjects) is not big, thus statistical power might be an issue. The sample size in preceding related studies is, however, similarly small (e.g., Dana et al., 2007; Andreoni and Bernheim, 2009). Hence, the effect sizes seem big enough for such samples.

Our experiment identifies shame as a channel that is behind increased giving in public situations. Committing a moral transgression by not giving as much as is expected would result in experiencing shame. Anticipating these psychological consequences of selfish behavior results in a transfer that is deemed compliant with morally/socially accepted behavior. A similar channel one could think of is pride. By giving more than expected one would experience pride or prestige. The psychological scale we used, the GASP, does not include a measure of pride, and therefore, we cannot test this potential channel further. It appears plausible that pride has a positive effect on giving, especially in situations where individuals can stick out from the crowd, in a positive sense, instead of avoiding a potentially shameful experience. However, the results of Samek and Sheremeta (2014) do not indicate a “prestige” effect in a related setting, a public goods game.

Our implementation of uncertainty is just one way to reduce the role of self-image concerns. Other ways to introduce moral wiggle room exist (e.g., plausible deniability, delegation, and strategic ignorance), and self awareness can also be manipulated directly. It remains to be seen, to what extent reduced giving following other interventions is also explained by the erosion of the beliefs/guilt system.

Finally, our experimental design considers two treatments with an exposure to an audience, Obs and ObsID. Both result in higher average transfers than in the baseline but only the difference in ObsID is significant, and this seems to be rooted in the disposition to shame. Although a third party is informed about the transfer, it seems that it is the public identification that kicks off the processes that lead to significantly increased giving. Nevertheless, the results in Obs differ from those in the baseline. Hence, a distinct process—not based on the disposition to guilt—might have been triggered. Either way, for the exposure effect in social-signaling models, public identification, like in ObsID, appears to be necessary.

## Data Availability Statement

The raw data supporting the conclusions of this article will be made available by the authors, without undue reservation.

## Ethics Statement

The studies involving human participants were reviewed and approved by Max Planck Institute of Economics ethics committee. The participants provided their written informed consent to participate in this study.

## Author Contributions

The author confirms being the sole contributor of this work and has approved it for publication.

## Conflict of Interest

The author declares that the research was conducted in the absence of any commercial or financial relationships that could be construed as a potential conflict of interest.

## Footnotes

^1^Note that our study focuses on (behavioral) economics and the emergence and evolution of social preferences within this field. While topics like altruism, cooperation and pro-sociality have been discussed before in related disciplines, our starting point is due to the focus on behavioral economics.

^2^For guilt there are four scenarios with negative behavior-evaluations (NBE) and four scenarios with repair responses (REP). For shame, there are four scenarios with negative self-evaluations (NSE) and four scenarios for withdrawal responses (WIT). See Appendix B for details of the GASP questionnaire.

^3^A stream of research tests the robustness of beliefs' causal effect on behavior (e.g., Vanberg, 2008; Ellingsen et al., 2010; Bellemare et al., 2011; Costa-Gomes et al., 2014; Kawagoe and Narita, 2014; Khalmetski, 2016; Ederer and Stremitzer, 2017).

^4^A related stream of literature proposes preferences for following (personal/social) norms as an explanation for pro-sociality in anony mous one-shot situations, see Capraro and Perc (2021) for a review. Moral preference models (e.g., Bicchieri and Chavez, 2010; Krupka and Weber, 2013; Kimbrough and Vostroknutov, 2016) find empirical support in various experiments (e.g., Capraro and Rand, 2018; Tappin and Capraro, 2018; Capraro et al., 2019).

^5^See Exley (2015) and Regner and Matthey (2017) for related studies that employ uncertainty in order to introduce an excuse for selfish behavior. In the model of Tirole et al. (2016) uncertainty is one of several ways to create moral wiggle room which, in turn, the self can use as a narrative to legitimate a selfish action. Note that uncertainty could also be interpreted to have a positive effect on transfers. In the self-signaling model of Grossman (2015), uncertainty cheapens the expected cost of sending a pro-social signal and more giving is predicted. However, his experimental tests do not provide conclusive supporting evidence.

^6^In a related setting, Malmendier et al. (2014) analyze subjects' behavior when an “exit option” is introduced into a double dictator game. They find substantial sorting out in a positive reciprocity condition (about 30%) but less than in a neutral condition (50%).

^7^Uncertainty is also a feature in the design employed by, for instance, Charness and Dufwenberg (2006) or Bracht and Regner (2013). Their chance move means that even though the agent behaves pro-social still a bad outcome for the principal can occur. Instead, an opportunistic choice for sure results in a bad outcome for the principal and implies that the agent knows this leads to disappointment. Note that the effect of uncertainty in our manipulation is broader/stronger, since an opportunistic choice (sending a low amount) does not necessarily mean that the principal receives a low amount.

^8^See Table 4 in the Appendix C for summary statistics of the respective variables.

^9^While it is generally acknowledged that there are no systematic behavioral differences between strategy method and direct response (Brandts and Charness, 2011), some evidence exists that trustees behave less trustworthy when using the strategy method (Casari and Cason, 2009). See also Garćıa-Pola et al. (2020) for behavioral differences in a related setting.

^10^Following Baron and Kenny (1986), we, first, establish that there is a correlation between the trust dummy and the transfer (controlling for the treatment dummies and the SVO angle) and that the trust dummy and second-order beliefs are correlated. Moreover, second-order beliefs as well as the trust dummy are correlated with the transfer. See Appendix D for details.

^11^A reviewer pointed out that guilt and shame are culture-specific characteristics. In the vein of WEIRD (see Henrich et al., 2010), it is important to note that our results are based on a predominantly German-speaking student sample. They may not necessarily extend to non-WEIRD contexts.
