# Supplementary material for: What's Behind Image? Toward a Better Understanding of Image-Driven Behavior
Source: Front Psychol. 2021 Jun 9;12:614575. doi: 10.3389/fpsyg.2021.614575 (PMC8219908; doi:10.3389/fpsyg.2021.614575)
Supplement: Supplementary file 2 [file Data_Sheet_2.pdf]

Instructions

Welcome and thank you for participating! In this experiment you can earn money depending on your decisions and the decisions of the other participants. **Therefore, it is very important that you read the instructions carefully.**

Please note that you are not allowed to exchange any information with the other participants.
Also, it is not allowed to talk to other participants during the whole experiment. Whenever you have a question please raise your hand. We will come to your place and answer your question. Please never ask your question(s) aloud. In case you break these rules we will have to end the experiment. Please switch off your mobile phones now.

General procedure

The experiment will take around 60 minutes. It consists of three stages. In each stage you take decisions. The respective decision situations will also be explained on the computer screen.
Only **one of the three** stages will be picked randomly for payment and you will be paid according to the choices in this stage. The exact way your earnings will be determined is explained further down. Your earnings from this experiment depend on your decisions and possibly on the other participants' decisions.
All amounts in the decision situations are stated **in Euro**. The exact amount will be paid to you in cash at the end of the experiment. Additionally, you will receive 2.50 Euro for your participation in the experiment and 3 Euro for completing the survey.

After filling out a questionnaire the experiment will be finished and you will receive your payment.

Overview of the procedure:

- reading the instructions, answering control questions
- stage 1
- (instructions) for stage 2
- (instructions) for stage 3
- questionnaire
- payment and end of the experiment

Details of the experiment

In the experiment three participants are matched. They are labelled as participant X, participant Y and participant Z. Whether you are participant X, Y or Z will be determined randomly at the beginning of the experiment. Hence, it is important that you familiarize yourself **with all roles**. The decision situation will **be played only once,** that is, there is only one round.

Decision situation

Only participants X and Y have a choice to make, Z does not. First participant X takes a decision. He/she can select either „left“ or „right“.

- The choice of „left“ results directly in a payoff of 5 EURO for participant X and 5 EURO for participant Y.
- If participant X chooses „right“, the payoffs of both participants will be determined by participant Y. 20 EURO are available to Y and he/she can decide, how to split this amount among X and Y. Y can select any transfer of a full EURO amount: 0, 1, 2, …, 18, 19, 20. If Y sends 5, then Y keeps 15 and X receives 5. If Y sends 15, then Y keeps 5 and X receives 15.

Participant Z does not take a decision and the choices of X and Y do not have any consequences on the payoff of Z. **Participant Z is informed though, which transfer Y selected. Moreover, Z is informed about the cabin number of participant Y.**

The following diagram illustrates the game and the possible payoffs:

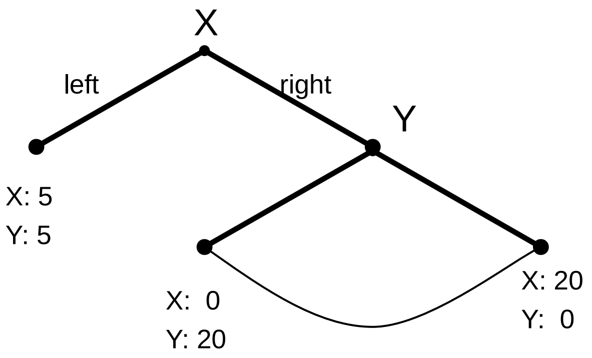

In the experiment participant Y will always be asked for their choice, independently of whether participant A has chosen „left“ or „right“.

Information about choices

You will only be informed about choices of the other participants at the end of the experiment, that is, after stage 3. Participant X gets to know which transfer participant Y has chosen (in case X selected „right“). Participant Y gets to know, whether X has chosen

„left“ or „right“. **Participant Z gets to know the choice of Y and also the cabin number of Y.** For receiving the payoff at the end of the experiment participants will be individually called to the front. For this purpose the cabin number of the participant is announced. The order of receiving the payoff (first cabin number 1 and 30 at the end, first number 30 and 1 at the end, or starting with cabin number 15 respectively 16 and then descending/ascending) will be determined randomly.
Thus independently of your cabin number you as participant Y may well be seen by the participant Z of your group.

Estimates

All participants, that is, also participant Z, will be asked for estimates in the experiment:

- Your estimate with respect to the decision of another participant in your group
- Your estimate about the belief of another participant in your group with respect to a decision

You can earn money with these estimates (per estimate up to 1 EURO). **The closer your estimate is to the actual value, the more money you earn.**

When entering your estimate you have the possibility to express how certain you are about your estimate. You can distribute a probability mass of 100 on the possible decisions.
In the experiment the possible decisions, for instance, the full numbers from 0 to 20 in the case of Y's transfer, are represented by intervals. If you are completely sure, then you would allocate the entire probability mass of 100 to respective interval. You can also spread your estimate on several estimates, though. This way you can express uncertainty about your estimate and you can extend your estimate to a broader area. Consider that the probabilities have to sum to 100.

The figure shows participant X's screen to enter the estimate with respect to the transfer of participant Y. The possible transfers of 0 to 20 are aggregated to 10 intervals. The expression [0,2) means that in this interval a transfer of 0 is included, as well as a transfer of 1, but a transfer of 2 is not included anymore (it is included in the intervall [2,4)).
At the lower end of the screen you are informed about the sum of the currently entered probability mass.

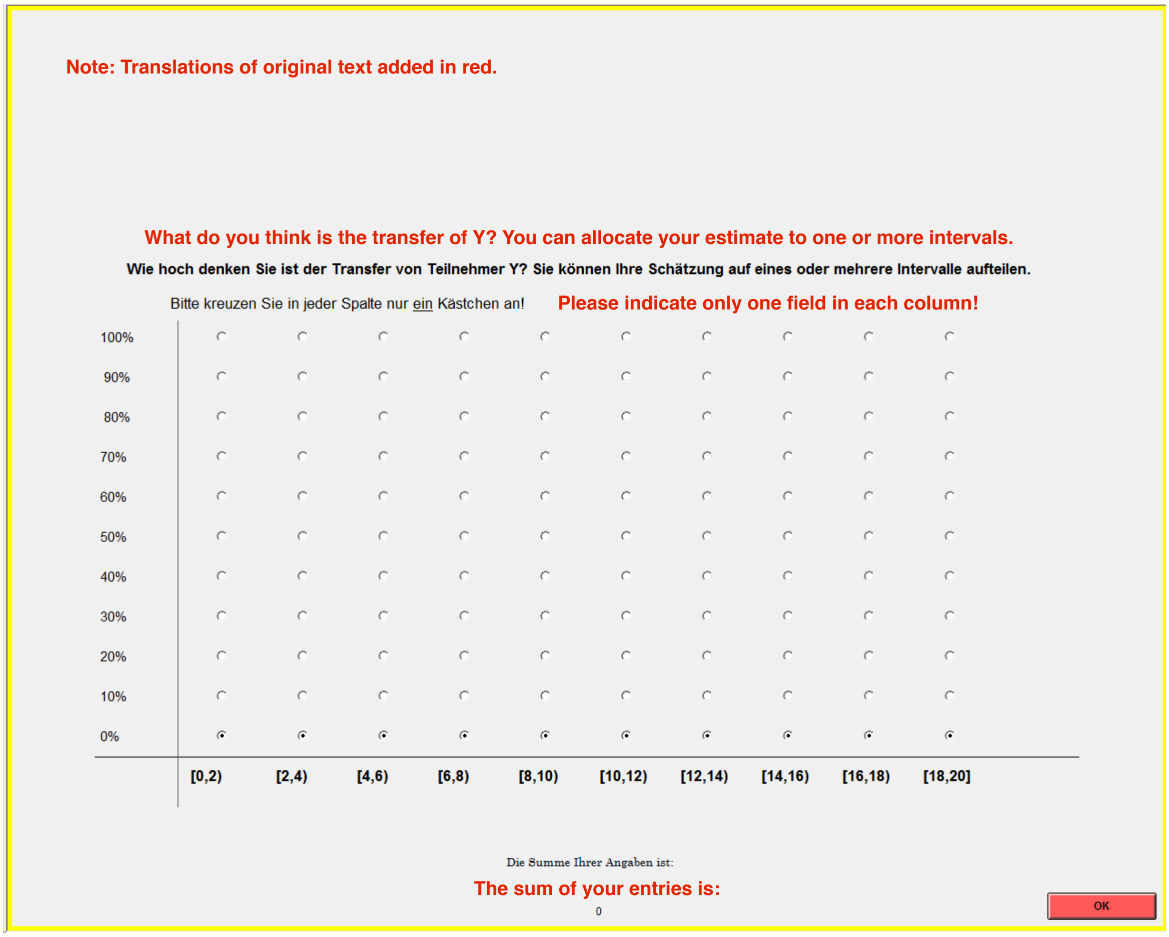

Example

You are the bouncer of a bar. Your boss will arrive at 23h and will want to know the share of female guests. Before 23h 100 persons entered the bar.

You paid close attention and you know that 47 men and 53 women were among the guests. In order to get the maximum payoff you should allocate the entire probability mass of 100 in the interval that contains the value 53. **In the experiment you will, of course, not know for sure what the share respectively the transfer is – you are supposed to estimate it.** Basically, it works the same, though. There will be control questions to practice entering estimates and testing your understanding.

Payoff

Your earnings from these estimates depends on how close your estimates are to the actual observed values in the experiment. The closer your estimate is to the real value, the more you earn. **The maximum possible earning is 1 EURO per estimate.** Participant X and Y make two estimates, participant Z makes four estimates.

Either way, it is optimal for you to disclose your actual estimates. On request we will show you (after the experiment), how exactly the payoff from estimates is computed.

Your earnings from the experiment

At the end of the experiment you will be notified about your payoff in the respective stages and which stage was randomly selected to be relevant for the earnings. You will receive your earnings in cash directly after the experiment is over, that is, after filling in the questionnaire.
